# Recent Advances in Transition-Metal-Catalyzed, Directed Aryl C–H/N–H Cross-Coupling Reactions

**DOI:** 10.1055/s-0036-1588536

**Published:** 2017-08-28

**Authors:** Martyn C. Henry, Mohamed A. B. Mostafa, Andrew Sutherland

**Affiliations:** WestCHEM, School of Chemistry, The Joseph Black Building, University of GlasgowGlasgow, G12 8QQUnited KingdomAndrew.Sutherland@glasgow.ac.uk

**Keywords:** amination, amines, amides, cross-coupling, dehydrogenation, transition metals

## Abstract

Amination and amidation of aryl compounds using a transition-metal-catalyzed cross-coupling reaction typically involves prefunctionalization or preoxidation of either partner. In recent years, a new class of transition-metal-catalyzed cross-dehydrogenative coupling reaction has been developed for the direct formation of aryl C–N bonds. This short review highlights the substantial progress made for *ortho* -C–N bond formation via transition-metal-catalyzed chelation-directed aryl C–H activation and gives an overview of the challenges that remain for directed *meta* - and *para* -selective reactions.

1 Introduction

2 Intramolecular C–N Cross-Dehydrogenative Coupling

2.1 Nitrogen Functionality as Both Coupling Partner and Directing Group

2.2 Chelating-Group-Directed Intramolecular C–N Bond Formation

3 Intermolecular C–N Cross-Dehydrogenative Coupling

3.1 *ortho* -C–N Bond Formation

3.1.1 Copper-Catalyzed Reactions

3.1.2 Other Transition-Metal-Catalyzed Reactions

3.2 *meta* - and *para* -C–N Bond Formation

4 C–N Cross-Dehydrogenative Coupling of Acidic C–H Bonds

5 Conclusions

## 1 Introduction

**Martyn C. Henry:**
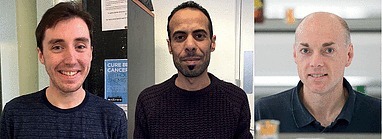
(left) was born in 1993 in Livingston, Scotland. He graduated with a 1st class M.Sc. degree in chemistry from Heriot-Watt University in 2016, while carrying out a placement year with Dr. Christopher Levy at Kansas State University and a final-year project under the supervision of Dr. Stephen Mansell on novel nickel complexes for olefin polymerization catalysis. Since 2016, he has been part of the Sutherland group at the University of Glasgow working toward his Ph.D., which is focused on the development of new transition-metal-catalyzed cross-coupling reactions. **Mohamed A. B. Mostafa** (middle) graduated with a B.Sc. degree (with high distinction) in chemistry from the University of Omar Al-Mukhtar, Libya, in 2003. In 2012, he was awarded an M.Sc. degree from the University of Wollongong, Australia, while carrying out a research project under the supervision of Professor Paul Keller on the stereoselective synthesis of chiral biaryl natural products. Since 2014, he has been part of the Sutherland group at the University of Glasgow, working toward his Ph.D. on the development of metal-catalyzed, one-pot multi-reaction processes for the preparation of polycyclic scaffolds. **Andrew Sutherland** (right) was born in 1972 in Wick, Scotland. After graduating with a 1st class B.Sc. Honours degree in chemistry at the University of Edinburgh in 1994, he undertook a Ph.D. at the University of Bristol under the supervision of Professor Christine Willis. This was followed by postdoctoral studies with Professor John Vederas at the University of Alberta and Professor Timothy Gallagher at the University of Bristol. In January 2003, he was appointed to a lectureship in the School of Chemistry at the University of Glasgow and currently holds the position of Reader. His research group’s interests are on the discovery of new transition-metal-catalyzed methodology and the development of radionuclide- and fluorescent-based molecular imaging agents.

The ubiquitous presence of aryl C–N bonds in natural products, pharmaceuticals, agrochemicals and organic materials has resulted in a wide range of methods for the efficient and selective synthesis of this motif. 1 Traditionally, aryl C–N bonds were formed using a combination of electrophilic aromatic nitration, followed by reduction of the resulting nitrobenzene. 2 However, with the development of catalytic bond-forming reactions (Scheme 1 ), the use of copper- or palladium-catalyzed amination of aryl (pseudo)-halides using Ullmann–Goldberg, Chan–Evans–Lam or Buchwald–Hartwig protocols have superseded the harsh conditions of the nitration/reduction approach. 3 4 More recently, the surge in methods for transition-metal-catalyzed C(sp ^2^ )-H activation has resulted in novel approaches for aryl C–N bond formation. 5 Initially, these novel transformations involved coupling of nucleophilic-like metalated intermediates with electrophilic or activated aminating reagents. However, methods have now been established that allow direct cross-dehydrogenative coupling (CDC) between aryl C–H bonds and non-activated amines and amides (Scheme 1 ). 5 This short review highlights the various methods that have been reported for both intramolecular and intermolecular aryl C–N bond formation using the cross-dehydrogenative coupling approach. In particular, highly regioselective *ortho* -amination and amidation through transition-metal-catalyzed chelation-directed activation of aryl C–H bonds will be described, as well as C–N bond formation from more reactive acidic aryl C–H bonds. The various one-pot strategies that have been reported for *para* -amination and amidation using activating groups are also discussed.


**Scheme 1** Transition-metal-mediated C–N bond-forming processes


## 2 Intramolecular C–N Cross-Dehydrogenative Coupling

### 2.1 Nitrogen Functionality as Both Coupling Partner and Directing Group

The first example of an oxidative cross-dehydrogenative coupling process was reported by Buchwald and co-workers for the preparation of unsymmetrical *N* -acylcarbazoles (Scheme 2 ). 6 The palladium-catalyzed process used oxygen and copper acetate as oxidants and allowed the efficient preparation of a range of *N* -acylcarbazoles bearing various functional groups and substituent patterns. Importantly, the *N* -acetamide coupling partner also acted as the directing group, which was necessary to overcome the energy barrier associated with the final C(sp ^2^ )–N reductive elimination step.


**Scheme 2** Synthesis of *N* -acylcarbazoles using a palladium-catalyzed CDC process


Other palladium(II)-catalyzed syntheses of carbazoles using intramolecular C–N bond-forming reactions have been reported. Work by the Gaunt 7 and Youn 8 research groups showed that the use of oxidants such as PhI(OAc) _2_ or Oxone permit C–N bond formation at ambient temperature. These studies also extended the substrate scope, demonstrating that *N* -alkyl and *N* -sulfonamide groups could be used as coupling partners. A similar cross-dehydrogenative coupling process of 2-aminobiphenyls using iridium(III) catalysis has allowed the synthesis of *N* –H carbazoles. 9 Again the nitrogen functionality is used as both the coupling partner and the directing group (Scheme 3 ). Following amine complexation of the iridium(III) catalyst and C–H activation, reductive elimination then gave the carbazole product. The resulting iridium(I) species was oxidized back to iridium(III) using a copper co-catalyst in the presence of air.


**Scheme 3** Iridium(III)-catalyzed synthesis of *N* –H carbazoles


This general approach for intramolecular C–N bond formation has been utilized for the preparation of a range of N-heterocycle-fused arenes. For example, copper-catalyzed protocols have been developed for the preparation of benzimidazoles 10 and pyrido[1,2- *a* ]benzimidazoles, 11 while palladium(II) catalysis has been used for the synthesis of 2-oxoindoles and 2-quinolinones. 12 13 For the palladium(II)-catalyzed synthesis of 2-oxoindoles (and 3,4-dihydroquinolinones), Yu and co-workers used *N* -methoxamides as the intramolecular N-coupling partner and for activation of the C–H bond (Scheme 4 ). 12 Although a CuCl _2_ /AgOAc system was used for re-oxidation of the palladium catalyst, resulting in relatively high temperature reactions, the process was found to have a broad scope, yielding the products in high yields. Furthermore, the *N* -methoxy groups were readily cleaved using either H _2_ /Pd/C or SmI _2_ , allowing efficient access to the parent lactam.


**Scheme 4** Palladium(II)-catalyzed synthesis of 2-oxoindoles


A lower-temperature intramolecular, palladium(II)-catalyzed­ C–N amidation process involving biaryl hydrazones was reported for the synthesis of 3-arylindazoles. 14 For unsymmetrical benzophenone tosylhydrazones, the regioselectivity of C–H activation and subsequent cyclization was controlled by electronic factors. For example, aryl rings bearing electron-donating substituents accelerated C–H activation resulting in cyclization on that particular ring (e.g., Scheme 5 ). Electron-withdrawing substituents instead deactivated C–H activation, and cyclization was favored for the more electron-rich unsubstituted benzene ring.


**Scheme 5** Palladium(II)-catalyzed synthesis of 3-arylindazoles


### 2.2 Chelating-Group-Directed Intramolecular C–N Bond Formation

Intramolecular C–N bond-forming reactions that use the nitrogen nucleophile to direct C–H activation do allow the efficient generation of cyclic products, however, due to strong coordination to the transition-metal catalyst, forcing conditions are generally required. In recent years, lower catalyst loadings and milder conditions have been achieved by using a chelating group to assist and direct transition-metal-catalyzed C–H activation. Several excellent methods for chelating-group-directed, copper-catalyzed intramolecular C–N coupling have been described, 15 16 but the majority of procedures have utilized palladium catalysis.

In 2012, the groups of Chen and Daugulis reported the directed palladium-catalyzed activation of C(sp ^2^ )–H bonds for intramolecular C–N bond formation. 17 18 Using PhI(OAc) _2_ as the oxidant and picolinamide as the directing group, a range of N-heterocycles was formed using low catalyst loadings and mild conditions (Scheme 6 ). The mild nature of these transformations was exemplified by the successful cyclization of substrates bearing labile functionality, such as aryl C–I bonds and the application of this process for the synthesis of complex natural products. 19


**Scheme 6** Picolinamide-assisted intramolecular C–N bond formation


More recently, Chen and co-workers extended this process for the challenging synthesis of benzazetidines. 20 Crucial for the success of this process was the use of a novel oxidizing agent, phenyliodonium dimethyl malonate [PhI(DMM)]. Computational studies with this reagent suggested a Pd(II)/Pd(III) intermediate with a bridging dimethyl malonate ligand as the resting state before reductive elimination. It was proposed that the rigidity of this intermediate would suppress the competing C–O bond-forming reaction and allow the kinetically controlled reductive elimination, leading to the strained ring products. This theory was confirmed by selective benzazetidine formation, in contrast to the outcome with the standard oxidizing agent, PhI(OAc) _2_ (Scheme 7 ). Interestingly, the cyclization of *ortho­* -arylbenzylamine substrates with PhI(DMM) favored formation of benzazetidines over six-membered C–N cyclization­ products.

As well as picolinamide, a range of N-directing groups has been used for palladium-catalyzed intramolecular C–N bond-forming reactions. 21 22 23 24 25 Directing groups such as 2-pyridinesulfonyl, 21 1-benzyl-1,2,3-triazole-4-carboxylic acid, 22 and oxalyl amides 24 are all effective chelating groups for N-heterocycle synthesis (Scheme 8 ). In particular, the *N* , *O* -bidentate oxalyl amide auxiliary, used in combination with hexafluoroisopropanol (HFIP) allowed the highly efficient synthesis of indoline products under mild conditions (60 °C). 24


**Scheme 7** PhI(DMM)-mediated benzazetidine synthesis



**Scheme 8** Indoline synthesis using various directing groups


The scope of the oxalyl amide directing group was further demonstrated with application for the efficient synthesis of six-membered N-heterocycles. 24 Using low palladium catalyst loading and PhI(OAc) _2_ as the oxidant, β-aryl­ethylamines and 2-phenoxyethylamines were cyclized under mild conditions to give the corresponding tetrahydroquinolines and benzomorpholines in good yields (Scheme 9 ). The process was tolerant of a wide range of aryl substituents including Br and I.


**Scheme 9** Oxalyl amide directed synthesis of tetrahydroquinolines and benzomorpholines


Modification of the oxalyl amide directing group by Zhao and co-workers for a simpler glycine dimethylamide motif, led to a system which could be used in palladium-catalyzed cascade processes (Scheme 10 ). 25 One-pot sequential β-C(sp ^3^ )–H arylation and intramolecular aryl C–H amidation processes were developed for the synthesis of a diverse set of 2-quinolinones. Glycine dimethylamide protected 2-phthalimidopropionic acids were excellent substrates for the palladium-catalyzed β-C(sp ^3^ )–H arylation with a range of aryl iodides. On completion of this step of the one-pot process, addition of PhI(OAc) _2_ facilitated the second palladium-catalyzed reaction, a cross-dehydrogenative coupling to give the quinolinone ring in high overall yields. Removal of the dimethylamide directing group was demonstrated under acidic conditions for the synthesis of the parent 2-tetrahydroquinolinones.


**Scheme 10** 2-Quinolinone synthesis using a palladium-catalyzed cascade process


## 3 Intermolecular C–N Cross-Dehydrogenative Coupling

### 3.1 *ortho* -C–N Bond Formation

The more challenging intermolecular C–H amination and amidation reactions, which generally require higher catalyst loading and reaction temperatures, have also required activation via a directing group. As a consequence, these processes only provide the *ortho* -aminated products. While a wide range of transition-metal complexes have been used to effect these transformations, the majority of processes developed have utilized copper catalysis.

#### 3.1.1 Copper-Catalyzed Reactions

In 2006, the groups of Yu and Chatani reported the first transition-metal-mediated intermolecular C–H amidation reactions. 26 27 Both processes used pyridine as the directing group and the difficulty of this transformation was demonstrated with the requirement of stoichiometric amounts of copper acetate and forcing conditions (Scheme 11 ).


**Scheme 11** Copper-mediated intermolecular C–H amidation of 2-phenylpyridine


Since these seminal reports, other groups have sought to develop catalytic methods for the cross-dehydrogenative coupling of 2-arylpyridines with non-activated amines and amides. While catalytic methods have been reported, these still require strong oxidants such as *tert* -butyl peroxide, 28 or the use of high temperatures. 29 30 31 For example, John and Nicholas showed that a range of nitrogen nucleophiles including sulfonamides, amides and anilines could be coupled with 2-phenylpyridine with only 20 mol% of copper acetate (Scheme 12 ). 29 However, the reactions required high temperatures (160 °C) and long reaction times (48 h). The modest yield obtained for the coupling reaction with 4-nitroaniline represents the difficulty of transition-metal-catalyzed intermolecular C–N bond formation with strongly coordinating amines.


**Scheme 12** Copper-catalyzed intermolecular C–H amination and amidation of 2-phenylpyridine


The mechanism of copper(II)-catalyzed aryl C–N bond formation is not well understood but catalytic cycles have been proposed (Scheme 13 ). 29 The first step involves complexation between 2-phenylpyridine and the copper catalyst, with C–H amidation occurring either via a single-electron­ process or by electrophilic aromatic substitution. It is generally believed that following ligand exchange of the acetate with the nitrogen nucleophile, a one-electron oxidation occurs to give a copper(III) intermediate, which can then undergo C–N reductive elimination to give the product. Aerobic oxidation of the resulting copper(I) acetate then regenerates the copper(II) acetate and completes the catalytic cycle.


**Scheme 13** Proposed catalytic cycle for the copper-mediated intermolecular C–H amidation of 2-phenylpyridine


Other directing groups, such as the 8-aminoquinoline auxiliary, have been investigated for copper-mediated cross-dehydrogenative couplings, which have improved both the catalyst loading and the reaction conditions. 32 33 34 The most general procedure was reported by Daugulis and co-workers, who showed that a wide range of secondary amines could be coupled with 8-aminoquinoline-derived benzamides with catalyst loadings as low as 10 mol% and at reaction temperatures of typically 110 °C (Scheme 14 ). 32


**Scheme 14** Copper-catalyzed intermolecular C–H amination of benz­amides using the 8-aminoquinoline directing group


Milder conditions have been achieved by further modification of both the aryl substituent and directing group. 35 36 37 38 In particular, the use of anilines in combination with the picolinamide directing group led to room-temperature C–H aminations. 36 In this study, Chen and co-workers reasoned that *N* -picolinamide-substituted anilines would readily undergo *ortho* -amination due to the formation of a six-membered­ O-ligated metallacycle (Scheme 15 ). In the event that a single electron transfer pathway was responsible for the transformation, a similar compact conformation could also be rationalized. Copper-catalyzed amination at room temperature using PhI(OAc) _2_ as the oxidant and magnesium chloride as an additive was complete after four hours and allowed the high-yielding amination of a wide range of amino­-substituted aromatic compounds. In general, a variety­ of functional groups attached to the aniline component were tolerated (e.g., OBn, NHBoc, halogens), while six-membered secondary amines gave the highest yields. By comparison, pyrrolidine was found to be unreactive (~5% yield) under these conditions.


**Scheme 15** Room-temperature copper-catalyzed intermolecular C–H amination of anilines using the picolinamide directing group


#### 3.1.2 Other Transition-Metal-Catalyzed Reactions

Around the same time as the first reported copper-catalyzed­ intermolecular C–N amidations, Yu and Che demonstrated that this could also be done using palladium catalysis (Scheme 16 ). 39 Unlike analogous copper-catalyzed amidations of 2-phenylpyridines that required high catalyst loading and forcing conditions, this procedure was shown to proceed with relatively low catalyst loading and moderate temperatures (80 °C). In the same report, aryl-substituted *O* -methyl oximes were also *ortho* -amidated in excellent yields under similarly mild conditions.


**Scheme 16** Palladium-catalyzed intermolecular C–H amidation of 2-phenylpyridines


Since this key breakthrough, other groups have investigated the use of various N-heterocycles or amine-based directing groups in combination with rhodium, 40 41 42 ruthenium 43,44 and palladium catalysts 45 for *ortho* -directed amidation. For example, Su and co-workers reported the application of rhodium(III) catalysis under low catalyst loadings and mild conditions for *ortho* -directed amidation using sulfonamides (Scheme 17 ). 40 As well as pyridines, a range of N-heterocycles, such as isoquinolines, pyrazoles and oxazolines could be used as the directing group.


**Scheme 17** Rhodium-catalyzed intermolecular aryl C–N bond formation with sulfonamides


More recently, Harrity and co-workers have expanded the scope of oxazoline-directed, rhodium-catalyzed *ortho* -C–H amidation and demonstrated that the products of this process could be used for the synthesis of 4-aminoquinazolines and quinazolinones (Scheme 18 ). 41 42 The rhodium(III)-catalyzed cross-dehydrogenative couplings with trifluoroacetamide were found to occur efficiently at temperatures as low as 25 °C. The resulting aryl trifluoroamides were hydrolyzed at room temperature and the subsequent crude anilines were then reacted with formamidine acetate effecting cyclization to give 4-aminoquinazolines. These were easily hydrolyzed to the corresponding quinazolinones under acidic conditions as shown with the preparation of the quinazolinone core of the drug candidate halofuginone­ (Scheme 18 ). This approach was further exemplified with the efficient synthesis of erlotinib, a 4-aminoquinazoline-containing tyrosine kinase inhibitor.


**Scheme 18** Rhodium-catalyzed intermolecular aryl C–N bond formation for the synthesis of 4-aminoquinazolines and quinazolinones


As well as N-heterocycle-directed C–N bond-forming reactions, various procedures have been developed using carbonyl-based auxiliaries. 46 In particular, benzamide derivatives have been widely used as substrates in combination with cobalt, 47 48 nickel 49 and iridium catalysts for C–H amination coupling reactions. 50,51 The development of efficient metal-catalyzed cross-dehydrogenative coupling reactions with strongly coordinating amines has been challenging. Niu and Song reported the use of cobalt(II) catalysis for the amination of 2-benzamidopyridine 1-oxides with alkylamines (Scheme 19 ). 47 Silver nitrate was found to be the most effective oxidant and in combination with the bidentate *N* , *O* -directing group, resulted in efficient cross-coupling­ with various six-membered cyclic secondary amines. However, in a similar fashion to some of the copper­-catalyzed C–N bond-forming processes, the use of pyrrolidine or non-cyclic secondary amines led to inefficient reactions.


**Scheme 19** Cobalt-catalyzed intermolecular C–H amination of 2-benz­amidopyridine 1-oxides



**Scheme 20** Iridium-catalyzed intermolecular C–H amination of *N* -adamantylbenzamides with electron-deficient anilines


Chang and co-workers have used sterically encumbered *N* -adamantylbenzamides for the iridium(III)-catalyzed, room-temperature cross-dehydrogenative coupling with anilines (Scheme 20 ). 50 It was proposed that the use of the bulky *N* -alkyl group would facilitate formation of the iridacyclic intermediate. On identification of the optimal oxidant­ (AgNTf _2_ ) and acetate additive [Cu(OAc) _2_ ], the iridium(III)-catalyzed aminations with a wide range of electron-deficient anilines were found to proceed in high yield. Electron-rich or *N* -alkyl variants were found to be unreactive for this process. Further work by the Chang group led to the development of an analogous iridium(III)-catalyzed amination of benzamides with primary alkylamines. 51 Secondary amines were completely inactive as coupling partners in this process due to the involvement of a nitrene insertion pathway.

Many of the limitations associated with general metal-catalyzed cross-dehydrogenative couplings with strongly coordinating amines such as electron-rich anilines, as well as secondary amines have recently been overcome using a ligand-promoted rhodium(III)-catalyzed activation process. 52 Coordination of 2-methylquinoline with the metal center of Cp*RhCl was found to accelerate C–H bond cleavage during amination of *N* -pentafluorophenylbenzamides (Scheme 21 ). The scope of the C–N coupling was found to be general for a wide range of amines. In particular, secondary amines, electron-rich and *N* -alkyl anilines were all found to couple efficiently with a broad range of substituted benzamides.


**Scheme 21** Ligand-promoted, rhodium(III)-catalyzed intermolecular C–H amination of *N* -pentafluorophenylbenzamides


Based on the substrate scope, control experiments, kinetic isotope measurements and the characterization of a rhodacycle intermediate by X-ray crystallography, a mechanism was proposed for this coupling (Scheme 22 ). 52 The pathway begins with complexation of 2-methylquinoline with the rhodium(III) species followed by C–H activation of the *N* -pentafluorophenylbenzamide to give the rhodacycle intermediate. Ligand exchange with the amine and reductive elimination generates the *ortho* -aminated product. The resulting rhodium(I) species is then oxidized back to Rh(III) by silver carbonate to complete the catalyst cycle. This study represents the current state of the art for transition-metal-catalyzed aryl C–H/C–N coupling reactions with amines.


**Scheme 22** Proposed catalytic cycle for rhodium(III)-catalyzed intermolecular C–H amination


### 3.2 *meta* - and *para* -C–N Bond Formation

Activation of *meta* -C–H bonds and subsequent cross-dehydrogenative­ coupling with non-activated amines and amides is not known. The only directed *meta* -C–H amination process, reported by Yu and co-workers, uses activated amines. 53 This approach combines directed *ortho* -palladation with Catellani’s norbornene-mediated relay process, 54 for an overall *meta* -functionalized product. This transformation has been used for the efficient *meta* -amination of anilines, phenols and benzylamines with a range of *O* -benzoyl hydroxylamines (Scheme 23 ).


**Scheme 23** Directed *meta* -amination using activated amines


The use of cross-dehydrogenative couplings for *para* -amination and amidation is similarly sparse. Again, reactions using activated nitrogen coupling partners are known. For example, Zhang and co-workers reported highly regioselective, palladium-catalyzed, *para* -directed amidation of bulky 2-methoxyphenyl amides using *N* -fluorobenzenesulfonamide. 55

A few metal-catalyzed C–H/N–H coupling reactions that generate *para* -aminated products have been reported, however­, there are limitations associated with these transformations­. Hartwig and co-workers have described a palladium-catalyzed intermolecular cross-dehydrogenative coupling of arenes with phthalimide. 56 The regiochemistry of the reaction was controlled by steric effects, but produced mixtures of *meta* - and *para* - *N* -aryl phthalimides. The regiochemistry of aryl C–H/N–H couplings with phthalimides has been improved using gold(I) catalysis. 57 The reaction is proposed to proceed by oxidation of the Au(I) catalyst to Au(III), which then undergoes electrophilic aromatic metalation with the arene. Following transmetalation with a phthalimide-derived iodane, reductive elimination forms the new C–N bond. This approach has allowed the preparation of a wide range of *para* -substituted-phthalimide-protected anilines (Scheme 24 ). However, eight equivalents of the oxidant PhI(OAc) _2_ are required for maximum yields.


**Scheme 24** Gold-catalyzed *para* -amidation


Another drawback of both of these procedures is the requirement of the reactions to be run in neat arene. 56 57 This limits the scope of these transformations to relatively simple aryl substrates. Metal-catalyzed *para* -directed amidations have been reported that use the aryl coupling partner as the limiting reagent. Wang and co-workers reported the one-pot, two-step synthesis of succinimide-protected anilines using gold(III) catalysis. 58 This process involves gold-catalyzed C–H activation to initially form the *para* -brominated intermediate (Scheme 25 ). The addition of copper then promotes an Ullmann–Goldberg-type coupling with the residual succinimide, yielding protected anilines in high yields as single regioisomers.


**Scheme 25** Gold(III)-catalyzed synthesis of *p* -succinimide-protected anilines


This approach of converting aryl C–H bonds into C–N bonds via an initial oxidation process before then implementing a copper-catalyzed coupling with a nitrogen nucleophile for the preparation of *para* -aminated aryl compounds has also been described by the Suna and Sutherland research groups. 59 60 Suna and co-workers showed that electron-rich arenes can be activated by hypervalent iodonium reagents to form unsymmetrical diaryl-λ ^3^ -iodanes, which were then smoothly coupled with amines using copper catalysis. 59 Sutherland and co-workers have used sequential iron and copper catalysis for the one-pot, two-step C–H/N–H coupling of anisoles, phenols, anilines and acetanilide-type aryl compounds with nitrogen nucleophiles (Scheme 26 ). 60 The *para* -C–H bond was initially brominated by iron(III) triflimide activation of NBS, via an electrophilic aromatic substitution reaction. Copper-catalyzed coupling with nitrogen nucleophiles such as N-heterocycles, amides and sulfonamides then completed the one-pot process. While both approaches are efficient and highly regioselective for the generation of *para* -substituted products, this type of one-pot cross-dehydrogenative coupling is restricted to electron-rich arenes.


**Scheme 26** One-pot C–H/N–H coupling using sequential iron and copper catalysis


## 4 C–N Cross-Dehydrogenative Coupling of Acidic C–H Bonds

The other key approach to regioselective aryl C–N bond formation is the activation and coupling of acidic C–H bonds with amines or amides. This was first reported in 2009 when the groups of Mori and Schreiber described the copper-catalyzed cross-dehydrogenative coupling of the C-2 position of azoles with both amines and amides. 61 62 Under aerobic conditions, the coupling was found to be efficient and general for a wide range of heteroaromatic compounds (Scheme 27 ). 61


**Scheme 27** Copper-catalyzed amination of azoles


The proposed mechanism for this transformation involves the deprotonation of the acidic C-2 hydrogen atom, followed by formation of an organocopper intermediate (Scheme 28 ). 61 62 Deprotonation of the nitrogen nucleo­phile, ligand exchange and reductive elimination then generates the coupled product. The catalytic cycle is completed by regeneration of the active copper species by oxidation with oxygen.


**Scheme 28** Proposed mechanism for the copper-catalyzed amination of azoles


Since these initial reports, a number of subsequent papers have described variants of this transformation, including alternative nitrogen coupling partners and an intramolecular version. 63 64 65 66 67 For example, Hirano, Miura and co-workers reported a one-pot copper-catalyzed C–H/N–H coupling and annulation reaction of azoles with *ortho* -alkynylanilines for the synthesis of *N* -azolylindoles (Scheme 29 ). 65 The domino sequence again used oxygen as the sole oxidant and was applied to the preparation of pharmaceutically relevant heterocyclic scaffolds.


**Scheme 29** Copper-catalyzed domino reaction for the synthesis of *N* -azolylindoles


While copper is most commonly used in the C–N cross-dehydrogenative coupling reaction of azoles, other transition metals such as cobalt, manganese and nickel have also been used for this transformation. 68 69 70 For example, cobalt was particularly effective for the amination of benzoxazoles with secondary amines at low catalyst loading and under mild conditions (Scheme 30 ). 68 In the same study, primary amines were coupled with benzoxazoles at slightly elevated temperatures (70 °C) using manganese(II) acetate as the catalyst. 68


**Scheme 30** Cobalt-catalyzed amination of benzoxazoles


As well as using azole-based acidic C–H bonds, a number of other acidic aryl C–H bonds have been utilized in C–N cross-dehydrogenative couplings. Schreiber, 62 Miura 63 and Su 71 have shown that polyfluoroarenes can undergo copper-catalyzed C–N bond coupling reactions with a wide range of nitrogen nucleophiles. For example, Su and co-workers­ used a combination of oxygen and TEMPO as the oxidant for the copper acetate catalyzed coupling of polyfluorobenzenes with electron-deficient anilines (Scheme 31 ). 71 Using a range of polyfluorobenzenes, they found that the coupling reaction was highly dependent on the acidity of the C–H bond.


**Scheme 31** Copper-catalyzed amination of polyfluorobenzenes


Copper-catalyzed amination and amidation of acidic C–H bonds has been extended to the selective C-2 substitution of quinoline *N* -oxides. 72 73 74 Under relatively mild conditions, reactions have been developed for coupling with lactams, oxazolidin-2-one (Scheme 32 ), secondary amines 72 73 and sulfoximines. 74 Following the coupling reaction, the quinoline *N* -oxides can be readily reduced to the parent quinoline using phosphorus trichloride. Interestingly, pyridine *N* -oxides are not substrates for this transformation.


**Scheme 32** Copper-catalyzed coupling of quinoline *N* -oxide with oxazolidin-2-one


Zhang and co-workers have shown that activation of quinolines to the corresponding *N* -oxides for C–N bond cross-dehydrogenative coupling reactions can be circumvented using Selectfluor as the oxidant. 75 Following optimization studies, which identified potassium carbonate as the most efficient base and nitromethane as the optimal solvent, an examination of the scope demonstrated that a wide range of substituted quinolines could be directly coupled with N-heterocycles (Scheme 33 ). Unlike *N* -oxide substrates, simple pyridines and other N-heterocycles could undergo C-2 coupling using this procedure. Based on control and kinetic isotope experiments, a C-2-fluorination followed by an S _N_ Ar mechanism was ruled out. Instead, the authors proposed a reductive elimination pathway via a Cu(III) species.


**Scheme 33** Direct copper-catalyzed selective C-2 amination of heterocycles


## 5 Conclusions

As described in this short review, a number of different strategies and procedures have now been developed that allow transition-metal-catalyzed directed C–N cross-dehydrogenative coupling of aryl C–H bonds with a range of non-activated amines and amides. The majority of early transformations used copper or palladium catalysis in combination with oxidizing agents such as PhI(OAc) _2_ . Recently, the development of more advanced methods, with the tuning of catalytic activity and using other transition-metal complexes such as those of cobalt, iridium or rhodium have resulted in milder conditions and transformations with broader scope. Many methods are now known for efficient aryl C–N bond formation through chelation-directed *ortho* -C–H activation or via activated acidic C–H bonds. However, while a few approaches for *para* -C–N bond formation have been reported using one-pot, transition-metal-catalyzed cross-dehydrogenative-like couplings directed by activating groups, there is still a need for additional methods that permit the highly regioselective *para* -amination of electron-deficient arenes. Furthermore, we still await the first transition-metal-catalyzed *meta* -directed C–H/N–H coupling reaction.
